# Targeting the Innate Immune System as Immunotherapy for Acute Myeloid Leukemia

**DOI:** 10.3389/fonc.2015.00083

**Published:** 2015-04-09

**Authors:** Emily Curran, Leticia Corrales, Justin Kline

**Affiliations:** ^1^Department of Medicine, University of Chicago, Chicago, IL, USA; ^2^Department of Pathology, University of Chicago, Chicago, IL, USA; ^3^Committee on Immunology, University of Chicago, Chicago, IL, USA; ^4^University of Chicago Comprehensive Cancer Center, Chicago, IL, USA

**Keywords:** acute myeloid leukemia, innate immune system, toll-like receptors, calreticulin, type I interferon, STAT3

## Abstract

Because of its disseminated nature and lack of tumor-draining lymph nodes, acute myeloid leukemia (AML) likely employs unique immune evasion strategies as compared to solid malignancies. Targeting these unique mechanisms may result in improved immunotherapeutic approaches. Emerging data suggest that a specific dendritic cell (DC) subset, CD8α DCs, may be responsible for mediating tolerance in AML and thus targeting the innate immune system may be of benefit in this disease. Promising immune targets include the toll-like receptors, calreticulin/CD47, the stimulator of interferon genes pathway, and signal transducer and activator of transcription 3 (STAT3). However, it is becoming clear that compensatory mechanisms may limit the efficacy of these agents alone and thus rationale combinations of immunotherapies are warranted. This review discusses the potential immune evasion strategies in AML, as well as discussion of the promising innate immune targets, both alone and in combination, for this disease.

## Introduction

Over 18,000 people will be diagnosed with acute myeloid leukemia (AML) in the United States this year (1). While the majority of patients will achieve a complete remission with standard induction chemotherapy (2), the relapse rate is high and over half will ultimately succumb to the disease. Despite an improved understanding of the molecular pathways altered in AML, no significant therapeutic advances have been achieved in many years. Allogeneic stem cell transplantation can be curative for some patients with AML (3), thought to be secondary to therapeutic graft-versus-leukemia effects resulting from donor-derived T cell recognition of minor histocompatibility antigens expressed on host leukemia cells (3–6). Unfortunately, only a minority of patients are candidates for stem cell transplantation, but the success of this procedure suggests that other immune strategies may be beneficial in AML.

It is known that AML cells express leukemia-associated antigens (LAA) that can be recognized by the immune system, including those derived from proteins such as proteinase 3 (PR3), receptor for hyaluronic acid-mediated motility (RHAMM), and Wilm’s tumor-1 (WT1), among others (7, 8). Objective clinical responses have been documented in patients with myeloid malignancies following PR3 and WT1 peptide vaccination (8, 9) and with cellular-based WT1 vaccines (10). These observations argue that vaccine-based immunotherapy for AML may be an effective strategy to reduce the risk of disease relapse, particularly those in a minimal residual disease state after remission induction and consolidation chemotherapy. However, there is limited data at this point to support this hypothesis. Further, many years of experience with cancer vaccine approaches for solid tumors has been ripe with failures, including dozens of therapeutic vaccination studies, which demonstrated minimal clinical efficacy (11).

It is clear that the development of cancer immunotherapies for hematologic malignancies, including AML, has lagged behind that for solid tumors. In part, this may be due to the availability of relatively effective chemotherapies and allogeneic stem cell transplantation for AML patients, thus limiting the interest in cultivating immune-based therapies in the field. Further, recent results of AML exome and genome sequencing has revealed a lower mutational burden in AML compared to most other cancers, such as melanoma and lung carcinoma, which impacts the number of leukemia-specific antigens (LSA) available for discovery and targeting (12, 13). While a number of LAA, including those mentioned above, have been identified in AML, they are also typically expressed in other tissues, including the thymus. Developing thymocytes capable of recognizing LAA with high affinity are likely deleted via central tolerance mechanisms, leaving behind low affinity T cells, which upon antigen encounter would be expected to elicit a weak and ineffective immune response.

Yet another barrier to generating effective immunotherapy for AML is that of immune evasion. Similar to solid malignancies, AML activates mechanisms in the host to avoid its immune-mediated elimination. Several of these negative regulatory mechanisms appear to be shared between solid and hematological cancers, including expression of negative costimulatory ligands, such as programed death-ligand 1 (PD-L1) and galectin 9 (Gal-9) on AML cells, and induced expansion of immunoregulatory cells, such as regulatory T cells (Tregs) and myeloid-derived suppressor cells (MDSCs) (14–18). However, recent work from our laboratory has demonstrated that AML may also promote unique immune evasion pathways not previously described in solid tumor settings (described below). Further understanding and exploration of the mechanisms of through which AML regulates the host immune system is expected to culminate in the development of effective immune therapies for this disease, as has been the case in solid tumors (19–21).

## The Role of Innate Immunity in Generating Immune Tolerance to AML

Our laboratory has recently characterized a novel and potent pathway through which the host innate immune system generates a T cell tolerant state in an animal AML model. In animals with AML, leukemia-specific CD8^+^ T cells underwent abortive proliferation and were deleted from the host. A small number of surviving antigen-specific T cells were partially dysfunctional and produced low levels of effector cytokines upon restimulation *ex vivo*, which suggested that they may have been anergized following antigen encounter. Deletional T cell tolerance in AML-bearing hosts appeared to be regulated by host antigen presenting cells (APCs) as it could be reversed *in vivo* following the administration of an agonistic anti-CD40 antibody, which resulted in enhanced anti-leukemia T cell immunity and prolonged survival (22).

More recent observations from our laboratory have suggested that a subset of host dendritic cells (DCs), called CD8α^+^ DCs, may mediate T cell tolerance in hosts with AML. Experiments in which fluorescently labeled AML cells were inoculated into mice revealed that CD8α^+^ DCs were uniquely capable of engulfing AML cells *in vivo* and of cross-presenting AML cell-derived antigens to T cells *ex vivo* (23). These results support a critical role for CD8α^+^ DCs in the immune recognition of AML. These data are important because they suggest that immune tolerance to AML may be initiated at the level of the innate immune system.

**Figure 1:**
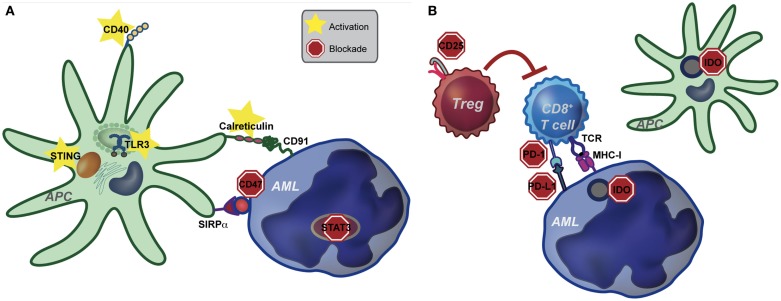
**Pathways of potential immune targeting in AML**. **(A)** Innate immunity may be targeted in AML through activation of the STING, TLR (specifically TLR-3) and CD40 receptors. Strategies to enhance calreticulin exposure on AML cells, combined with CD47-blocking antibodies may also be effective. Lastly, STAT3 signaling appears to inhibit the innate immune response, including hampering the effect of TLR9, and inhibition of STAT3 signaling in AML cells leads to differentiation to antigen presenting cells (APCs) with an activated DC phenotype. Activation of the innate immune system, either by stimulating activating pathways or blocking inhibitory pathways, ultimately leads to increased IFN-γ production by CD8^+^ T cells. **(B)** Increased IFN-γ resulting from activating of the innate immune system may lead to compensatory up-regulation of other immune evasion pathways, such as PD-L1, indoleamine-2,3-dioxygenase (IDO), and regulatory T cells (Treg). While innate immune activation will be critical to induce anti-leukemia T cell priming, combination therapy targeting compensatory pathways will be key in eliciting a clinically significant anti-leukemia immune response.

The ability of DCs to activate T cells is dependent upon their “activation state.” In the absence of inflammatory stimuli (i.e., under steady-state conditions), DCs are quiescent and are important in this context to maintain peripheral tolerance to self-antigens. Conversely, in the solid tumor context, danger-associated molecular patterns (DAMPs) released by dying cancer cells are sensed by DCs, leading to enhanced antigen presentation, as well as increased expression of costimulatory ligands, chemokines, and cytokines. These changes effectively license DCs to prime a functional anti-tumor T cell response. Although speculative, we believe that due to the disseminated nature of AML, as well as the lack of a classical tumor-draining lymph node, DCs, which engulf and cross-present AML-derived antigens, may not be exposed to sufficient “danger signals” from AML cells to mediate their licensing. The net result is the induction of T cell tolerance to AML. If, in fact, innate immune cells are central to tolerance induction in leukemia-bearing hosts, then targeted activation of innate immunity may be sufficient to overcome tolerance and promote clinically meaningful immunity against AML. In the following sections, we will discuss several innate immune pathways that are amenable to targeting in order to enhance immunity in hosts with AML, as well as the potential for combination therapy (see Figure 1).

### Toll-like receptors

The toll-like receptors (TLRs) are a family of transmembrane receptors, which recognize highly conserved microbial structures (i.e., bacterial cell wall components, CpG DNA, viral nucleic acids), termed pathogen-associated molecular patterns (PAMPs). Activation of TLR signal transduction pathways leads to induction of inflammatory cytokines, chemokines, MHC, and costimulatory molecules (24). There are at least 10 TLRs in humans and several, including TLR3, have been targeted as immunotherapy for cancer.

The natural TLR3 ligand is double-stranded RNA. TLR3 stimulation results in the activation of the transcription factors interferon regulatory factor 3 (IRF3) and NF-κB through the adaptor molecule TRIF, culminating in interferon (IFN)-β production (25). Unlike the majority of TLRs, TLR3 signals in a MyD88-independent manner (24). Interestingly, TLR3 appears to be preferentially expressed on CD8α^+^ DCs (26, 27). Because this DC subset appears to be responsible for mediating tolerance to AML, at least in pre-clinical models, TLR3 may be a promising target for immunotherapy in leukemia. Polyriboinosinic polyribocytidylic acid [poly(I:C)] and a related compound, polyinosinic-polycytidylic acid-polylysine-carboxymethylcellulose (poly-ICLC), are TLR3 agonists, which have been utilized to target TLR3 both in pre-clinical and clinical studies. It has been shown that loading leukemia cells with poly(I:C) by electroporation, and thus mimicking a viral infection, leads to enhanced immunogenicity of leukemia cells, as well as DC maturation and activation (28). Thus, targeting TLR3, with the goal of activating host DCs to reverse T cell tolerance is a promising therapeutic strategy.

Poly(I:C) was initially investigated as monotherapy for hematologic and solid malignancies several decades ago, with disappointing results. Early clinical trials demonstrated that poly-ICLC was a potent inducer of type I interferon and, among 19 adult and pediatric patients with refractory solid tumors or acute leukemia treated, 1 child with acute lymphoblastic leukemia (ALL) had a complete remission (29). Adverse reactions in these trials included fever, nausea, hypotension, thrombocytopenia, leukopenia, erythema, polyarthralgia, and myalgia (29). In a phase II trial of children with acute leukemia and neuroblastoma, including 28 children with ALL, no complete responses were achieved and significant toxicity was observed, leading the authors to halt further study of this agent in other childhood tumors (30). Similarly, phase I studies of poly(I:C) and poly-ICLC in adults with melanoma, ovarian cancer and other advanced cancers demonstrated significant toxicity with few objective clinical responses (31–33). Newer formulations, lower doses, and intramuscular injections of poly(I:C) may decrease its toxicity (34, 35). Nevertheless, the systemic delivery of poly(I:C) as a single agent is not likely to have a major impact as an immunotherapy for cancer.

More recently, poly(I:C) has re-emerged as a viable cancer immunotherapeutic as a cancer vaccine adjuvant. Pre-clinical studies have demonstrated that, when administered as a single adjuvant to various tumor antigen vaccine formulations (including cell-based, peptide, protein, exome, or viral), poly(I:C), and poly-ICLC resulted in enhanced tumor-associated antigen specific and functional T cells, with reduced tumor growth (36). To date, clinical trials of poly(I:C) and poly-ICLC as cancer vaccine adjuvants have mainly been conducted in solid malignancies, specifically gliomas, but have shown promising results (36). A phase I/II trial of DCs loaded glioma-associated antigens and poly-ICLC adjuvant in 22 patients with recurrent malignant glioma showed progression-free survival of at least 12 months in over one-third of patients, as well as one complete response (37). Recent studies have also investigated the use of poly(I:C) as a vaccine adjuvant in combination with other immune stimulators, such as Monatide-ISA-51, granulocyte–macrophage colony-stimulating factor, or a TLR7/8 agonist (resquimod) (36). These combinations appear to be well-tolerated, even in pediatric patients (38).

Poly(I:C) is currently being investigated as an adjuvant to vaccine therapy in an early phase clinical trial in patients with AML in complete remission following chemotherapy or allogeneic stem cell transplantation (Table 1). This trial combines a WT1 peptide vaccine with basiliximab and either poly(I:C) or montanide ISA 51, with the goal of determining the side effects and best method of administering vaccine therapy. Another ongoing phase I trial combines poly(I:C) with the DEC-205/NY-ESO-1 fusion protein CDX-1401 and decitabine for treating patients with MDS or AML (Table 1). DEC-205 is an endocytic receptor expressed primarily by DCs, as well as thymic epithelial cells (39) and NY-ESO-1 is an immunogenic protein that is usually very low or absent in myeloid leukemias, but is expressed following hypomethylating agents (40). The DEC-205-NY-ESO-1 fusion protein, called CDX-1401, is a full length NY-ESO-1 protein sequence fused to a monoclonal antibody against DEC-205. A previous phase I trial of this agent in solid malignancies, combined with adjuvant TLR agonists, such as poly(I:C), demonstrated disease stabilization and regression in a subset of patients, with no dose-limiting toxicities (41). Thus, while poly(I:C) treatment may be of limited benefit as monotherapy, it has demonstrated moderate effectiveness when coupled with other immunotherapeutic approaches.

**Table 1:** **Select ongoing trials in AML/MDS**.

Immunotherapeutic mechanism	Drug	Combination	Disease	Primary outcome	Phase (Clinical trial #)
PD-1 blockade	CT-011	DC AML vaccine	AML, CR1, or CR2	Toxicity	Phase 2 (NCT01096602)
	Nivolumab	N/A	AML, CR1, or CR1i	PFS	Phase 2 (NCT02275533)
PD-L1 blockade	MK-3475	N/A	MDS	Toxicity, ORR	Phase 1b (NCT01953692)
	MEDI4736	N/A	MDS, following hypomethylating agents	Safety, toxicity	Phase 1 (NCT02117219)
CTLA-4 blockade	Ipilimumab	N/A	Relapsed/refractory AML/MDS	Toxicity, Treg percentage	Phase 1 (NCT01757639)
	Ipilimumab	N/A	Recurrent AML/MDS	Toxicity	Phase 1/1b (NCT01822509)
IDO inhibition	INCB024360	N/A	MDS	ORR	Phase 2 (NCT01822691)
TLR3 agonist	Poly-ICLC (compared to basilixumab)	WT1 peptide vaccine	AML, CR, or CRi	Peptide-specific immune response, Treg numbers	Phase 1 (NCT01842139)
	Poly-ICLC	DEC-205-NY-ESO-1 fusion protein, decitabine	AML with <30% blasts, MDS	Toxicity	Phase 1 (NCT01834248)

*MDS, myelodysplastic syndrome; CR, complete remission; CRi, complete remission with incomplete count recovery; CR1, first complete remission with incomplete count recovery; CR2, second complete remission; CR1i, first complete remission with incomplete count recovery; PFS, progression free survival; ORR, overall response rate; Treg, regulatory T cell (www.clinicaltrials.gov)*.

## Calreticulin and CD47

Calreticulin (CRT) is a chaperone protein that normally resides in the endoplasmic reticulum (ER), where it functions to ensure that misfolded proteins are not exported to the Golgi apparatus. However, more recent studies have demonstrated that CRT translocates from the ER to the cell-surface following cell stress or apoptosis (42). Upon translocation to the cell surface, CRT appears to stimulate phagocytosis by macrophages via their expression of low-density lipoprotein-receptor related protein (LRP), also known as CD91 (43).

Some chemotherapeutic agents (anthracyclines and oxaliplatin) and radiation have been shown to induce ER stress and promote CRT surface translocation in cancer cells, resulting in their recognition and phagocytosis by innate immune cells, such as macrophages and DCs (44). In turn, these innate immune cells become capable of priming antigen-specific T cell responses directed against malignant cells, termed “immunologic cell death.” Elevated cell surface CRT expression has also been observed on viable malignant cells – including leukemia blasts (45, 46), suggesting that cell death may not be a prerequisite for CRT translocation. However, the mechanistic link between CRT expression and its downstream effects on adaptive immunity has not been elucidated. Furthermore, using publicly available gene profiling data sets, Chao analyzed CRT mRNA levels in tumors from patients with a variety of cancers, including neuroblastoma, bladder cancer, and mantle cell lymphoma and found, perhaps counterintuitively, that elevated CRT expression correlated inversely with event-free and/or overall survival (45).

To directly investigate the role of CRT on immunity to AML, our lab has recently generated AML cells engineered to express high-levels of cell-surface CRT. Consistent with previous studies, CRT-expressing AML cells promoted enhanced anti-leukemia T cell responses and prolonged survival in mice. This effect was dependent on adaptive immunity, as it did not occur in Rag^−/−^ hosts, or following *in vivo* T cell depletion. Although a clear effect of CRT expression on *in vivo* phagocytosis by DCs or macrophages was not observed in our experiments, further studies are needed to clarify the mechanism through which CRT promotes anti-leukemia immunity.

Calreticulin-mediated phagocytosis of cancer cells can be inhibited by expression of anti-phagocytic proteins, such as integrin-associated protein (IAP), also known as CD47 (43, 45). CD47 is broadly expressed on all cells and transmits a “do not eat me” signal to phagocytes expressing its receptor, signal regulatory protein-α (SIRP-α) (47). Increased CD47 expression on human AML cells promotes their survival through evasion of phagocytosis (48), and blocking CD47 on human cancer cells in xenotransplantation models promotes their phagocytosis and elimination by innate immune cells (48–50). CD47 is more highly expressed in a subset of human acute lymphocytic leukemia (ALL) samples and is an independent predictor of survival and disease refractoriness (51). Pre-clinical studies of a blocking monoclonal antibody against CD47 enabled phagocytosis of ALL cells by macrophages *in vitro*, inhibited tumor engraftment *in vivo*, and eliminated ALL in mice engrafted with primary human ALL (51). In a non-Hodgkin lymphoma model, anti-CD47 antibodies were found to be synergistic with rituximab, leading to disease “cure” through Fc receptor-dependent and -independent stimulation of phagocytosis (52). This data suggest that calreticulin/CD47 targeting may be a beneficial immunotherapeutic strategy for AML as well. Phase I clinical trials utilizing CD47 antibodies are currently being developed (http://stemcell.stanford.edu/CD47/). Thus, CD47 over-expression on leukemia cells could represent yet another immune evasion mechanism, which is targetable through receptor blockade.

## Type I Interferon and STING

The importance of type I IFN signaling in the development of functional anti-tumor immunity has been well-characterized (53). Studies in bone marrow chimeric mice harboring autochthonous, carcinogen-induced sarcomas have revealed that type I IFN produced in response to a developing cancer must be “sensed” by host hematopoietic cells, rather than by malignant cells, in order for immune-mediated tumor rejection to ensue (54, 55). Type I IFN signaling, particularly by DCs, is critical to generate functional T cell responses that ultimately mediate tumor elimination (54, 55), and mice lacking the IFNα/β receptor (IFNAR) in DCs fail to reject immunogenic solid tumors (54). Within the DC compartment, CD8α^+^ DCs were required to respond to type I IFN and to prime anti-tumor T cell responses in a pre-clinical melanoma model, as Batf3^−/−^ mice, in which CD8α^+^ DCs fail to develop, generate very poor spontaneous anti-tumor T cell responses (55). Collectively, these data suggest a critical role for type I IFN in the host immune response to cancer and may be mediated by CD8α^+^ DCs.

Until recently, the tumor-derived signals that regulate type I IFN production in the tumor-bearing host were unknown. However, it has been recently demonstrated that cytosolic DNA from dying tumor cells may be a potent inducer of the type I IFN response, through the activation of a cytosolic DNA-sensing receptor called stimulator of interferon genes (STING) (56). STING was originally discovered to stimulate of production of type I interferon using expression screening of human and mouse cDNA transfected into T cells harboring a luciferase reporter under control of the IFNβ promoter (57). Subsequently, STING was found to be required for the induction of innate immune responses to cytosolic, (non-CpG) pathogenic DNA following viral infection (58). Recently, the ligands for STING have been identified as cyclic dinucleotides, known as cyclic-GMP-AMPs (cGAMPS), which are generated from GTP and ATP by the cytosolic enzyme cGMP-AMP synthase (cGAS). cGAMP binding to STING induces its homodimerization and trafficking from the ER to the Golgi (59, 60). In the Golgi, STING recruits tank binding kinase 1 (TBK1), resulting in its phosphorylation and activation of the transcription factor interferon regulatory factor-3 (IRF-3). IRF-3 then translocates to the nucleus and activates transcription of type I IFN (57, 58).

Administration of STING agonists to C1498 AML-bearing mice bearing results in significant expansion of functional leukemia antigen-specific T cells, as well as significantly improved survival (61). While further understanding of the mechanisms underlying the efficacy of STING agonists are warranted prior to human translation, these data suggest that the STING pathway, by stimulating IFN-β production in the host, may represent an effective therapeutic target for AML.

## Signal Transducer and Activator of Transcription 3

Signal transducer and activator of transcription 3 (STAT3) belongs to a family of seven cytoplasmic transcription factors, which mediate cellular growth, differentiation and apoptosis (62, 63). While a number of cytokines and growth factor receptors can activate STAT3, IL-6 signaling represents one key pathway. IL-6 signaling through GP130 and Janus kinases (JAKs) results in STAT3 phosphorylation, dimerization, and translocation to the nucleus, leading to further production of IL-6 (creating a feed-forward loop), and up-regulation of anti-apoptotic genes (63, 64). Other activators of STAT3 include IL-10, IL-23, and LPS activation of TLR4 and TLR9 (64).

Signal transducer and activator of transcription 3 has been shown to be aberrantly activated in a majority of cancers, most commonly secondary to activation of upstream kinases, such as epidermal growth factor receptor and platelet-derived growth factor receptor (64). Constitutive activation of STAT3 in malignant cells results from the loss of function of negative regulators, such as the suppressor of cytokine signaling (SOCS) family of proteins, protein tyrosine phosphatases (PTPs), the protein inhibitors of activated STATs (PIAS) family, and the ubiquitin-proteasome degradation pathway (62). Constitutive STAT3 activation in malignant cells promotes cell-cycle progression and prevents apoptosis through a variety of mechanisms, including p53 inhibition and up-regulation of genes such as cyclin D1/D2, MYC, and Bcl-XL (65). In pre-clinical models, constitutive STAT3 activation alone results in malignant transformation (65) and inhibition of STAT3 activity results in arrest of tumor development and apoptosis (62).

In addition to its intrinsic role in promoting transformation and cancer progression, active STAT3 also contributes to cancer immune evasion and has been implicated in infectious (such as *H. pylori* and Epstein–Barr virus) and non-infectious (such as colitis) inflammation-induced carcinogenesis (63, 64, 66). STAT3 activation antagonizes expression of anti-tumor T helper 1 cytokines (such as IL-12 and IFN-γ), mediates T regulatory cell expansion in tumors, and is required for the immunosuppressive effects of MDSCs and tumor-associated macrophages, as well as for the development of tumor-promoting TH17 T cells (64). STAT3 activity has also been found to hamper the effect of locally administered TLR9 ligands (67). Ablating STAT3 through use of inhibitors or knock-out mice results in enhanced function of DCs, T cells, natural killer cells, and neutrophils in tumor-bearing mice and induces growth inhibition of established tumors (68).

Signal transducer and activator of transcription 3 may also promote leukemogenesis, and active STAT3 has been observed in primary leukemia blasts in a majority of AML patients (62). Furthermore, STAT3 activation is correlated with inferior outcome in AML. In a study of 63 patients with AML, STAT3 activation, as determined by STAT3 phosphorylation on Western Blot and DNA-binding activity on electrophoretic mobility shift assay, was present in 44% of patients, where it was associated with a significantly decreased disease-free survival (median 20.6 versus 8.7 months) (69). Thus, STAT3 has become an attractive therapeutic target in AML. However, until recently, it was unclear whether the adverse effects of STAT3 activation in leukemia were a result of its anti-apoptotic and proliferative effects, or also a result of its immune modulatory effects.

In a pre-clinical study of a mouse model of Cbfb-MYH11/Mpl-induced leukemia, which mimics inv(16) AML in humans, systemic administration of CpG-STAT3 small interfering RNA (siRNA) resulted in eradication of established AML in mice (70). STAT3 inhibition failed to restrain leukemia progression in immunodeficient mice, demonstrating that the anti-leukemia effects of STAT3 inhibition were likely immune-dependent. Furthermore, targeted STAT3 blocking/TLR9 activation resulted in differentiation of AML blasts to APCs with an activated DC phenotype, increased the ratio of tumor-infiltrating CD8^+^ T cells to regulatory T cells, and promoted CD8^+^ T cell dependent regression of leukemia (70).

Current methods of inhibiting STAT3 are indirect, including inhibition of upstream tyrosine phosphorylation through tyrosine kinase inhibitors, activating negative regulators of STAT (including SOCS and PIAS), disruption of STAT nucleocytoplasmic shuttling, and blockade of cytokine/growth factor receptor binding (62). However, small molecule inhibitors are being developed to block STAT3 dimerization and transcriptional activity (71). While further study and development of STAT3 inhibitors are needed, based on the promising pre-clinical results and evidence of activation in AML, STAT3 inhibition warrants further evaluation in clinical trials in leukemia.

## Combination Therapies with Innate Immune Activation

While immune-based therapies, such as CTLA-4 and PD-1-blocking antibodies are highly effective in a subset of patients with melanoma and other solid cancers, monotherapy for most patients is not sufficient for complete or prolonged tumor eradication, likely due to compensatory immune evasion pathways activated in the cancer-bearing host (19, 20, 72, 73). Thus, it follows that targeting the innate immune system alone is unlikely to be a sufficient strategy to eliminate AML. In addition, as discussed below, the increased IFN-γ produced by innate immune activation leads to up-regulation of several compensatory immune evasion pathways, making combination therapy even more pertinent in this setting. We envision that the simultaneous manipulation of several non-redundant negative regulatory pathways, including negative costimulatory receptor (i.e., PD-1, CTLA-4, TIM-3, LAG-3) blockade, removal of suppressor cell populations, and targeting inhibitory enzymes (IDO, arginase), in combination with innate immune targeting may lead to synergistic effects. Although a detailed discussion of the other immune evasion pathways is beyond the scope of this review, a few promising targets will be discussed and are shown in Figure 1.

Programed cell death-1 (PD-1) is a negative regulatory receptor expressed on the surface of activated T cells, B cells, and NK cells and binds to PD-L1, which is expressed on various malignant cells, including AML cells. Zhang and colleagues have demonstrated that the ligand for PD-1, PD-L1 is up-regulated on a murine leukemia cell line *in vivo* and after *in vitro* exposure to IFN-γ (15). Other groups have also demonstrated that up-regulated expression of PD-L1 is dependent on exposure of tumor cells to IFN-γ produced by effector T cells (74). Because many of the therapies targeting innate immune activation result in T cell activation and IFN-γ production, targeting PD-L1 in combination with innate immune activation may be synergistic. Trials of PD-1 and PD-L1 inhibitors have demonstrated efficacy in solid tumors (21, 72), and clinical trials of PD-1 and PD-L1 inhibitors are currently underway in AML (Table 1). Future trials may consider combination of PD-1 or PD-L1 inhibitors with innate immune activators.

Inhibitory enzymes, such as indoleamine-2,3-dioxygenase (IDO), may also be beneficial synergistic targets with innate immune activators. IDO catalyzes tryptophan degradation, with tryptophan metabolites negatively regulating T cell activation and survival. Similar to PD-L1, IFN-γ production by CD8^+^ T cells has been shown to result in up-regulation of IDO (74). IDO inhibitors are currently being investigated in clinical trials in lymphoid malignancies and MDS (Table 1). IDO expression has been demonstrated in blasts of AML patients (75) and is correlated with decreased survival (76). Based on this data, trials in AML, including combination with innate immune activators are warranted.

Regulatory T cells (Tregs) are naturally occurring immunosuppressive CD4^+^ T cells, which are important in the maintenance of peripheral tolerance to self-antigens (77–79). Inhibition or depletion of Tregs results in enhanced anti-tumor T cell responses and control of tumor progression in transplantable cancer models (80, 81). In addition, Tregs accumulate in leukemia-bearing mice and depletion results in enhanced anti-leukemia T cell responses (16). CD8^+^ T cell infiltration is associated with recruitment of Tregs in the tumor microenvironment, making this an attractive immunotherapeutic target (74). In the clinical setting, Treg depletion has been demonstrated to be achievable in cancer patients, although responses have been conflicting (82–84). However, based on the evidence available, Treg depletion may be beneficial in combination with innate immune activation.

It is unlikely that targeting any single immune pathway will be sufficient for effective and prolonged treatment of AML. Because AML appears to rapidly induce tolerance of leukemia-specific T cells in a manner, which depends on the innate immune system, we envision that activation of the innate immune will be a key component of immune-based therapies and necessary in order for functional anti-leukemia T cell responses to be primed. However, it is likely that even functionally primed T cells will be subjected to additional negative regulation in the host and thus combination with other immune evasion targeted therapies will be important in order for clinically meaningful anti-leukemia immune responses to be raised in the host.

## Conclusion

The innate immune system appears to play a key role in immune evasion by AML and thus, activating innate immunity will likely be critical in immunotherapeutic strategies for this disease. Further insight into the unique mechanisms of immune evasion in AML is needed, but early data suggest promising immunotherapeutic approaches with TLR agonists, the calreticulin-CD47 pathway, STING activation, and STAT3 inhibition. However, because of compensatory immune evasion pathways, combination with other immune targets, such as PD-1/PD-L1, IDO inhibition, and Treg depletion will likely be needed. Given these findings, future studies may benefit from incorporating innate immune activation strategies and will hopefully lead to the same exciting results already demonstrated in solid malignancies.

## Author Contributions

EC and JK reviewed relevant literature. EC drafted the manuscript. JK revised the manuscript and supervised EC. LC assisted with manuscript revisions and designed the figures. All authors read and approved the final manuscript.

## Conflict of Interest Statement

The authors declare that the research was conducted in the absence of any commercial or financial relationships that could be construed as a potential conflict of interest.
